# Tissue-specific Gene Expression Changes Are Associated with Aging in Mice

**DOI:** 10.1016/j.gpb.2020.12.001

**Published:** 2020-12-11

**Authors:** Akash Srivastava, Emanuel Barth, Maria A. Ermolaeva, Madlen Guenther, Christiane Frahm, Manja Marz, Otto W. Witte

**Affiliations:** 1 Hans-Berger Department of Neurology, University Hospital Jena, Friedrich Schiller University Jena, 07747 Jena, Germany; 2 Bioinformatics/High Throughput Analysis, Friedrich Schiller University Jena, 07743 Jena, Germany; 3 FLI Leibniz Institute for Age Research, 07745 Jena, Germany

**Keywords:** Aging, RNA-seq analysis, Inflammaging, Electron transport chain, Tissue aging

## Abstract

**Aging** is a complex process that can be characterized by functional and cognitive decline in an individual. Aging can be assessed based on the functional capacity of vital organs and their intricate interactions with one another. Thus, the nature of aging can be described by focusing on a specific organ and an individual itself. However, to fully understand the complexity of aging, one must investigate not only a single tissue or biological process but also its complex interplay and interdependencies with other biological processes. Here, using RNA-seq, we monitored changes in the transcriptome during aging in four tissues (including brain, blood, skin and liver) in mice at 9 months, 15 months, and 24 months, with a final evaluation at the very old age of 30 months. We identified several genes and processes that were differentially regulated during aging in both tissue-dependent and tissue-independent manners. Most importantly, we found that the **electron transport chain** (ETC) of mitochondria was similarly affected at the transcriptome level in the four tissues during the aging process. We also identified the liver as the tissue showing the largest variety of differentially expressed genes (DEGs) over time. Lcn2 (Lipocalin-2) was found to be similarly regulated among all tissues, and its effect on longevity and survival was validated using its orthologue in *Caenorhabditis elegans*. Our study demonstrated that the molecular processes of aging are relatively subtle in their progress, and the aging process of every tissue depends on the tissue’s specialized function and environment. Hence, individual gene or process alone cannot be described as the key of aging in the whole organism.

## Introduction

Aging is a highly complex process characterized by progressive physiological changes throughout all organs and tissues. Intrinsic and extrinsic factors influencing life span or relating to aging-specific diseases have been examined widely in the past. It has become increasingly clear that aging has divergent effects on different tissues at both the gene expression and physiological levels [1], [2]. For example, the role of the liver during aging has been intensively discussed, and it is suggested that replicative senescence, exposure to toxins (such as drugs and free radicals), and diet play important roles in the process of liver aging [3], [4]. In 2015, Lans et al. [5] described how damage responses are altered uniquely for different tissues in *Caenorhabditis elegans* because of distinct DNA repair systems. Additionally, several studies have linked the aging process with an increase in inflammation, and suggested that changes in lymphoid organs also play an important role [6]. However, despite decades of research, aging remains an incompletely understood process.

One important approach to understanding the mechanisms of aging is to examine changes in the gene expression levels at different ages. An increase in the expression of inflammatory and stress response genes along with the accumulation of ubiquitinated proteins with age has been reported by many independent studies based on microarray or RNA-seq experiments. In particular, antigen processing and presentation, NF-κB signaling, and lipid metabolism, as well as complement and coagulation cascades, have been shown to be relevant to inflammation pathways during aging [1], [7], [8]. In addition to certain processes, some specific genes have also been shown to be associated with aging. Examples of such genes are: members of the cathepsin gene family, apolipoprotein genes, genes involved in the complement system, members of the Signal Transducer and Activator of Transcription (STAT) gene superfamily, and members of the Tumor Necrosis Factor Receptor (TNFR) gene superfamily [9], [10], [11].

Most of these studies focused on only one tissue (frequently the brain) and included a limited number of time points (often including a time point before maturation). Studies comparing gene expression changes during aging in multiple tissues across different age points are scarce. One exception is the extensive microarray-based study done by Jonker et al. [1] with five different organs and six age points. This study reported distinct aging signatures in different organs [1].

Here, we presented a comprehensive study of age-related transcriptional changes in four tissues of mice: brain, blood, liver, and skin. We generated a unique set of 92 RNA-seq transcriptome libraries from mice at four different ages: a young but already mature age of 9 months, an intermediate age of 15 months, an old age of 24 months, and the very old age of 30 months. Part of the brain data have been analyzed in a previous publication. Survival studies have shown that 72 % of C57BL/6 mice lived to an age of 24 months, while only 4% were long-lived and survived to an age of 30 months [12]. It is important to note that mentioned age groups are in relation to C57BL/6 male mice and may differ for other strains and sex.

Our study revealed tissue-specific and tissue-independent genes and processes that were differentially affected during aging. We identified seven genes that were differentially expressed in all four tissues during aging, along with genes encoding various subunits of the mitochondrial electron transport chain (ETC) that were regulated in a similar fashion among the analyzed tissues. While the number of differentially expressed genes (DEGs) and their molecular biological processes were comparable among the skin, brain and blood, the liver showed a much higher level of age-linked gene expression differences, making it an interesting candidate for future research.

## Results and discussion

### PCA shows characteristic regulation at the transcriptome level for every tissue during aging

**Figure 1:**
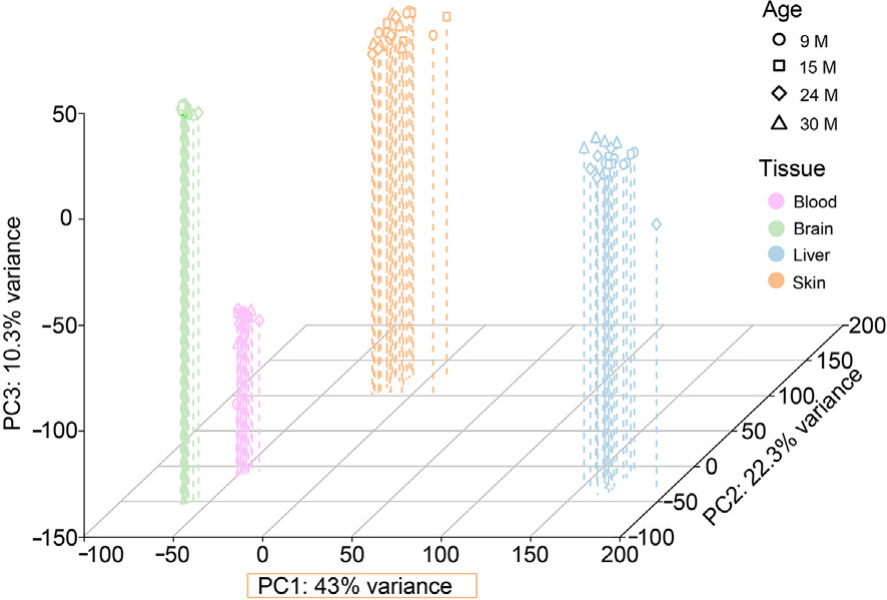
**Principal component clustering of RNA-seq libraries** The three-dimensional principle component analysis (PCA) plot of 92 RNA-seq libraries was generated based on the 750 most variable genes, showing the highest variance in expression between time points in all samples. While all libraries clustered very well together according to their tissues, no clear distinction regarding different age groups could be made. PCA plots of each tissue based on different sets of genes are available at https://osf.io/ru2sb/, showing in general always the same clustering.

To get a general overview of the 92 RNA-seq samples from the four tissues investigated, we performed a principle component analysis (PCA) based on the 750 genes that were most variable with age in all samples (Figure 1; for details visit https://osf.io/ru2sb/).

As expected, the sample replicates of the individual tissues (blood, brain, liver and skin) clustered together. Tissues are clearly distinguishable, indicating that tissue-specific genes are present among the 750 most variable genes.

Being most separated in the first principle component, the liver showed the largest differences from other tissues. In the liver and skin, we observed the highest dispersion of samples in the PCA plot, indicating broader expression variability at different time points. The blood and especially the brain replicates clustered more closely together. This is in agreement with the fact that the brain is a tissue that undergoes only minor changes once it is fully developed, despite some new insights which show plasticity in some specific areas [13]. However, a clear distinction of different ages within individual tissues was hardly possible with this approach (for details visit https://osf.io/ru2sb/), since age-dependent expression changes were subtle, and no larger set of obvious common aging-linked factors was present in these four tissues. Only for the liver could one observe measurable separation of the replicates of the young time point at 9 months and the old time point at 24 months from all other samples.

### Predicting important modulators of aging for each tissue

**Figure 2:**
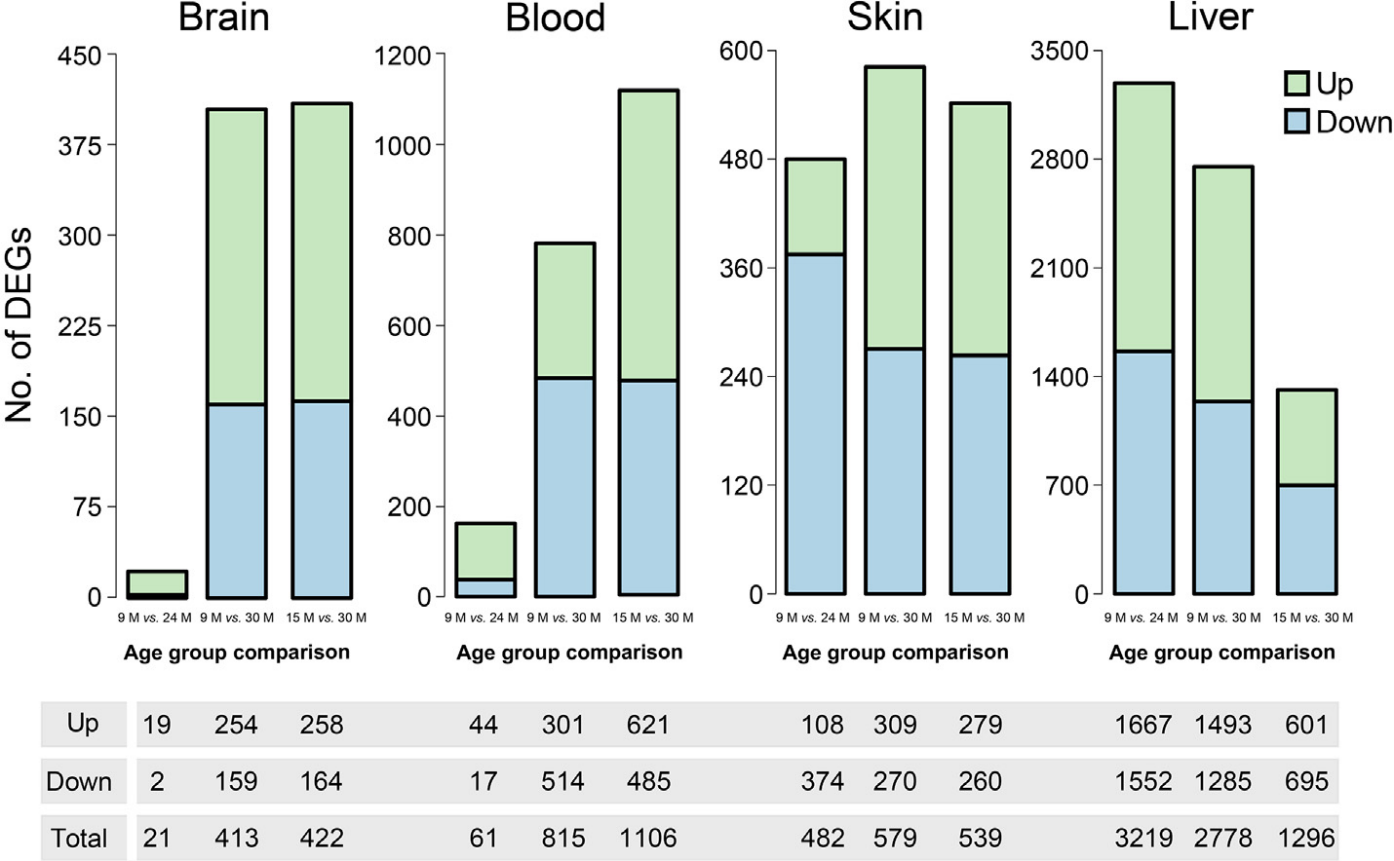
**Differentially expressed genes in mouse tissues during aging** The numbers of differentially expressed genes (DEGs) in different age group comparisons for each tissue were shown. Aging occurred as an accumulation of errors over time, manifesting as a high number of DEGs observed in the age group comparisons of 9 months *vs*. 30 months and 15 months *vs*. 30 months in the brain, blood, and skin. The liver did not follow this pattern and showed a high number of DEGs in every age group comparison. The scales of the y-axes of the plots are  different. More details about all age group comparisons and identified DEGs can be found in https://osf.io/ge8b3/.

In total, we identified 579, 1329, 1237, and 5185 genes that were differentially expressed in the brain, blood, skin, and liver, respectively, among the pairwise age group comparisons. Specific numbers of DEGs identified are given in Figure 2 (https://osf.io/k79tp/).

#### Aging patterns of gene expression in different tissues

Within the brain, consecutive pairwise age group comparisons showed only minor changes in gene expression, indicating that normal aging is a slowly progressive process at the gene expression level with subtle changes between close time points. However, a larger number of genes (~ 400) with significant changes were observed between wider time spans, especially those including the very old mice (9 months *vs*. 30 months and 15 months *vs*. 30 months). This shows the progressive nature of the aging process, which seems to accelerate at later time points.

A very similar pattern of gene expression changes was observed in blood and skin, both containing a generally higher number of affected genes. Skin showed more DEGs at an earlier time point (9 months *vs*. 24 months), which can be interpreted as a slightly accelerated aging process compared to brain and blood.

#### The brain shows more signs of inflammaging

In particular, the most significant DEGs in the brain were genes of the complement and immune systems, such as *C4a*, *C4b*, *Tlr2*, *Cst7*, and *Ifit3*. These included brain-specific ones, such as *Gfap*, which is known to act during development but is also a marker for activated astrocytes with aging [14]. All these genes have been previously reported to be up-regulated in the brain with age. While proactive immune functions can imply protection from infections, persistent low-grade inflammation can also be deleterious [15]. Thus, inflammaging might be one of the primary causes of aging in the brain. Additionally, we also observed moderate up-regulation of cell cycle regulators, *e.g.*, *Cebpa*, which is a common observation in aging neurons [16]. For details, visit https://osf.io/k79tp/.

#### The blood mainly shows markers of senescence due to replicative aging

We observed age-linked down-regulation of the hematopoietic cell growth and survival negative regulator *Rhoh*, which is known to be a strong inhibitor of NF-κB, and other general T and B cell migration and differentiation factors, such as *Chst3* [17]. In addition, regulators of various cancer pathways appeared to be differentially expressed: *Id3* and *Dusp2*, which hint at a potential cancer suppressing mechanism for very old mice, and *Dpep2*, which is known to play an anti-leukemia role [18], [19]. Thus, dysregulation of cell division and differentiation is highly prominent with aging. In addition, similar to that in the brain, the immune system was activated with aging in blood cells, and although it was mostly restricted to the up-regulation of immunoglobulin genes (*i.e.*, different *IgK* isotypes) from B cells, we observed up-regulation of other immune-related genes, such as *Sirpb1-a/b/c*, *Retnlg*, *Mpo*, and *Prg2* expressed by macrophages, monocytes, and eosinophils. In comparison with the very late age (at 30 months), we also found alteration of more general inflammation factors, such as *Clec4e*, *Ifitm2*, and *C4b*.

#### The skin shows a distinctive aging pattern due to environmental exposure

In the skin, a surprisingly large variety of transcription factor genes appeared among the topmost DEGs in the 9 months *vs*. 24 months comparison. We observed down-regulation of many genes associated with the extracellular matrix, such as different members of the collagen gene family and *Flnc* which encodes one of three filamins. A reduction of the extracellular matrix is already implicated with aging due to external factors such as UV radiation, and has been examined in a variety of studies [20], [21], [22]. However, no clear trend for affected molecular pathways was observed. Only in comparisons to the 30-month-old mice were some common patterns identified. We found important skin functions, such as tight junction, focal adhesion, and olfactory transduction, to be most significantly affected between 24 months and 30 months of age. This underlines the fact that a decline in tissue-specific functions can be seen with increasing age. When compared to the brain and blood, only a few immune- and inflammation-associated genes were found to be differentially regulated in the skin, indicating that the typical inflammaging signs may be less prominent in this tissue.

#### The liver shows several divergent processes related to aging

We identified the highest number of DEGs (5185) related to aging in the liver, making it the most dynamic of the investigated tissues. Young adult tissues (9 months and 15 months) mainly displayed changes in genes involved in drug metabolism and reactive oxygen species (ROS) management; in particular, these are mainly cytochrome genes. In addition, immune-related genes were highly significantly affected. Notably, inflammatory processes were regulated but showed no clear general activation or deactivation at these ages, with half of them being down-regulated (*e.g.*, *C8b* and *Ilr1*) and the other half being up-regulated (*e.g.*, *Tlr12* and *H2eb1*) (for details visit https://osf.io/ge8b3/).

With progressing age (9 months and 15 months compared to 24 months), more genes related to inflammation were up-regulated, pointing to an increased inflammation process. Additionally, cell cycle- and cell adhesion-related genes such as *cyclin D1* and *cadherin 1* were very significantly up-regulated in the 15 months *vs*. 24 months comparison. Mis-regulation of those genes is known to contribute to cancer progression, which is an important cause of death with aging [23]. This is consistent with our pathway analysis (see below), as cancer-promoting pathways increased between 9 months and 24 months of age. However, genes associated with cancer suppression did not undergo a significant change.

We observed the up-regulation of genes involved in the immune system among the topmost regulated genes in the very old mice compared to younger ones, mainly represented by the activation of immunoglobulin and interferon genes. The two most dramatically up-regulated genes here were *S100a8* and *S100a9*, which encode two proteins belonging to the S100 calcium-binding protein family that form a heterodimer and are known to play a major role in inflammation and tumorigenesis [24], [25].

When comparing the two oldest time points (24 months *vs*. 30 months), hardly any immune-related genes were found among the most significantly altered genes, which is in agreement with the inflammation processes being highly active in old age (24 months) and not likely to decrease or increase further with ongoing aging (30 months).

Instead, the most significant up-regulated DEGs are involved in cell death induction (*e.g.*, *Dedd2*) or directly related to cancer (*e.g.*, *Brap*) [26]. Interestingly, the autophagy-inducing gene *Atg2a* was up-regulated, although autophagy activity has been described as decreasing across all tissues during aging [27].

The number of DEGs greatly varied between the investigated tissues, being lowest in the brain (579) followed by the skin (1237), blood (1329), and liver (5185). This might be caused not only by aging, but also by the special roles of these tissues. The brain is the most protected tissue of all and practically stops growing after maturation, and it also shows only low regenerative capacities. Being the most exposed tissue of an individual, skin undergoes constant abrasion due to light, friction, and other environmental factors. These external stresses can cause the distinctive aging phenotype of skin over time. Interestingly, we found changes in the activation of genes involved in focal adhesion, tight junction, and olfactory in the comparison between the two oldest time points (24 months *vs*. 30 months). This could be explained by loss of function due to permanent exposure to environmental stresses. For blood, genes linked to cell division seemed to be the main signature of physiological changes of processes during aging. Blood constantly undergoes proliferation, unlike most tissues which normally undergo division only when they suffer damage. The liver is the most metabolically active tissue and involved in detoxification processes. Thus, the liver is constantly exposed to exogenous factors, undergoing constant regeneration and showing high regenerative potential. However, this extraordinary plasticity likely makes the liver more susceptible to inflammation and cancer development, as indicated by our data on age-linked gene expression changes.

### Clustering of KEGG pathways into categories reveals the most prominent processes affected in aging

#### Analysis of DEGs reveals tissue-specific processes with aging

**Figure 3:**
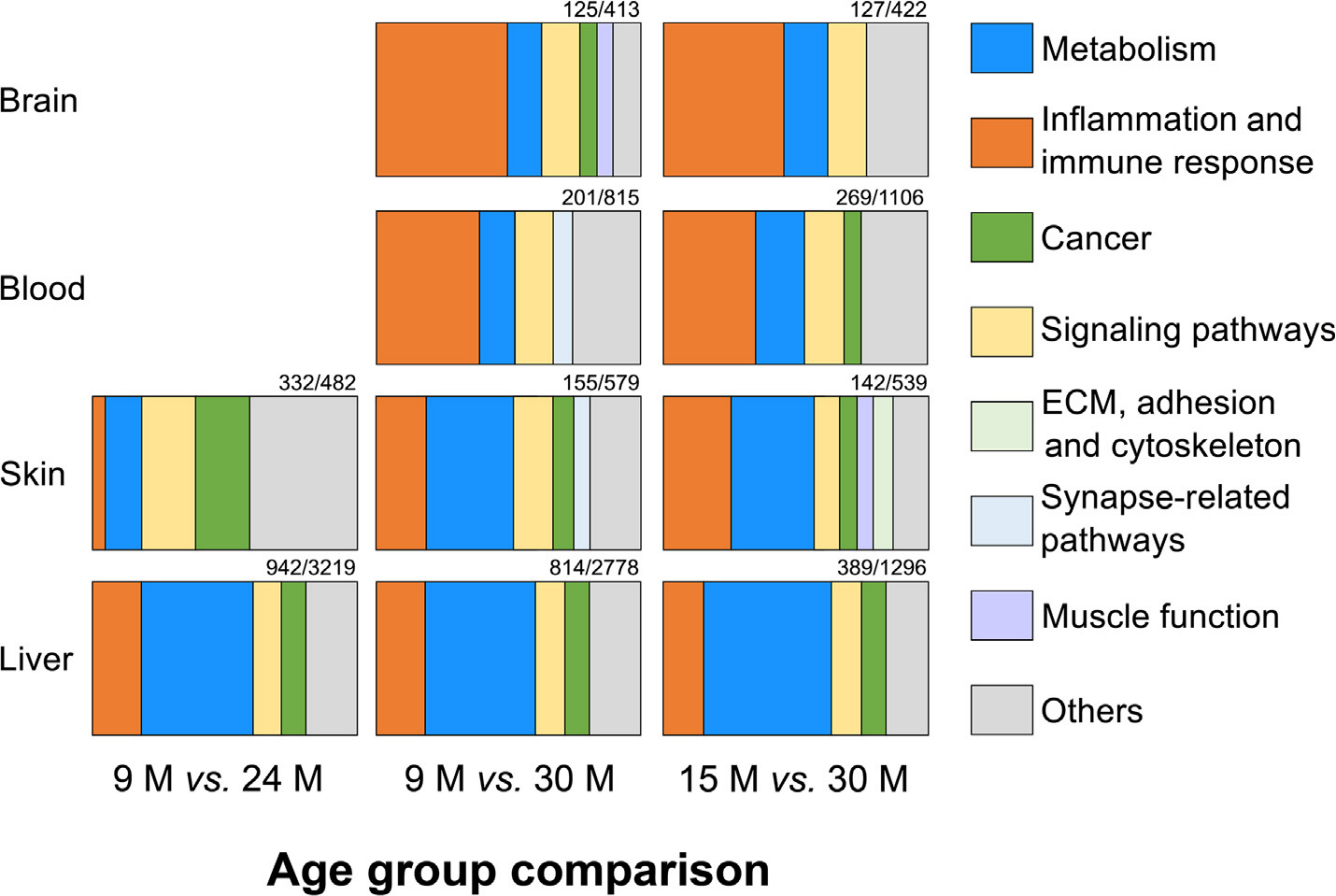
**Tissue-specific change of enriched pathways during aging** Kyoto Encyclopedia of Genes and Genomes (KEGG) pathway category enrichment analysis was performed based on DEGs (Figure 2) in different age group comparisons for each tissue. The number of annotated DEGs and the total number of DEGs are displayed at the top of each subplot. Any pathway contributing less than 5% of the DEGs is categorized as ‘Others’ (gray). Note that not all age group comparisons were included here, since they had too few DEGs for a sufficient pathway enrichment analysis. More details can be found in https://osf.io/k7dba/.

To analyze our data in a broader context, we enriched Kyoto Encyclopedia of Genes and Genomes (KEGG) pathways in different age group comparisons for each tissue using the Database for Annotation, Visualization and Integrated Discovery (DAVID, V6.8) (Figure 3; for details visit https://osf.io/h6dx3/).

#### Brain

In the brain tissue, when comparing 9 months and 15 months to 30 months of age, we found 413 and 422 genes to be differentially expressed, respectively. Among these DEGs only 125 and 127, respectively, were found to be annotated and were associated with a known pathway within the KEGG database. For the age group comparison of 9 months *vs*. 30 months, 50% of the DEGs were related to inflammation and immune response, 14.4% to signaling pathways, 12.8% to metabolism, 7.2% to cancer, and 5.6% to muscle function. In addition, 90% of DEGs were higher expressed at 30 months of age. Interestingly, in the age gruop comparison of 15 months *vs*. 30 months, we observed an almost 13% decline in inflammation and immune response compared with that in the age group comparison of 9 months *vs*. 30 months, while a similar degree of increase was observed in metabolism and signaling pathways. This result suggested that with progressive aging, there may be an increase in chronic inflammation, and hence, a lower number of DEGs and pathways could be annotated to the same category at later time points, while an increase in metabolism- and signaling-related pathways can be due to either dysregulation or an effort to adjust to the increased inflammation. Additionally, the contribution of cancer- and muscle function-related pathways dropped below 5%, putting them in the ‘Others’ category. This change indicated that pathways related to cancer are more tightly regulated at 9 months than at 15 months, and therefore, we cannot detect a significant difference when comparing later time points. This can be explained by the accumulation of damage with age [28], [29]. In the remaining DEGs (the ‘Others’ category), a relatively high number of DEGs (15) were related to the cell adhesion molecule pathway with a relative low *P* value (2E−6) for both age group comparisons. These factors can be prognostic markers for blood–brain barrier integrity.

Prolla [30] has already discussed similar results with respect to brain aging by comparing his findings with calorie restricted mice. He reported several up-regulated genes related to stress and immune response, along with an accumulation of ubiquitinated proteins in control mice. However, this effect was slightly reduced in calorie restricted mice.

#### Blood

In the blood tissue, we identified 815 and 1106 DEGs within the age group comparisons of 9 months *vs*. 30 months and 15 months *vs*. 30 months, respectively, of which 201 and 269 were annotated using DAVID. DEGs contributing to inflammation and immune response still made up the largest group in the significantly altered pathways at both age group comparisons. A closer analysis on the transcript expression revealed that DEGs related to inflammation were again higher expressed while DEGs related to the H2 complex (MHCII) were lower expressed at 30 months compared with those at 9 months and 15 months. Changes in metabolism-related pathways were on the same order as those observed in the brain, with only a slight increase to 13% and 18% at the 9 months and 15 months, respectively. The major difference between the brain and blood was the percentage of DEGs within the cancer pathways. It was less than 5% in the 9 months *vs*. 30 months comparison and thus part of the ‘Others’ category, but it was approximately 7% in the 15 months *vs*. 30 months comparison. In the later age group comparison, most of the DEGs related to cancer pathways were found to be higher expressed at the age of 30 months. These results suggested that in the blood, the changes related to cancer are more prominent at 15 months than those at 9 months and might increase at a later age.

#### Skin

The skin seems to age in a relatively distinctive manner, possibly due to direct exposure to external environmental factors, which is limited for most of the other tissues. For the 9 months *vs*. 24 months comparison, 482 DEGs were identified. Of these, 332 were annotated by DAVID, and 302 belonged to the olfactory transduction pathway with a very low enrichment *P* value of 7E−223. Skin has many different receptors, helping an individual to perceive its immediate environment [31]. All 302 DEGs contributing to olfactory transduction were lower expressed at 24 months of age, probably showing a loss in this specific ability when contrasted to young adult mice at 9 months. When comparing the mice at two younger ages (9 months and 15 months) to the 30-month-old ones, we identified approximately the same number of DEGs (579 and 539). Of these, 155 and 142 DEGs were associated with known KEGG pathways. Among both age group comparisons, changes related to metabolism and cancer were approximately equal, but there was an increase of 6% in inflammation- and immune response-related up-regulated genes, from 19.7% to 25.7%, with advanced age. This complements the reduction of signaling pathway-related genes from 14.8% to 9.7%. The most notable changes here were genes related to the muscle function and extra cellular matrix (ECM), adhesion, and cytoskeleton categories, which showed an 7% increase with a dysregulation of the primary functions of the skin (*e.g*., barrier function and wound healing). It was also complemented with a decreased expression of collagen-related genes. Additionally, within the signaling pathway category, we found that the PI3K-Akt and FoxO signaling pathways were most significantly changed. Both pathways are strongly involved in the processes of aging and contribute to longevity [32].

#### Liver

Further, in the liver, we identified 3219, 2778, and 1296 DEGs for the abovementioned age group comparisons, among which 942, 814, and 389 were categorized into associated known pathways, respectively. We observed the same trend in consecutive age group comparisons, *i.e.*, a decrease in inflammation and immune processes while an increase in metabolic pathways with age. Major gene expression changes were observed in the comparisons of 9 months *vs.* 15 months and 15 months *vs.* 24 months (for details visit https://osf.io/w3a28/). From 9 months to 30 months, most genes were higher expressed in the older mice, irrespective of their functional categories. Furthermore, changes in pathways associated with cancer and signaling were relatively constant. As the age progresses, more and more DEGs fall into mixed functional categories, showing a slow dysregulation with aging. In absolute terms, changes in the metabolic processes were by far the largest in the liver, highlighting the importance of this tissue in the regulation of these functions during aging.

#### Comparison of tissues

Most of the DEGs from the brain and blood were involved in inflammation and immune response, as we already suspected from their topmost regulated genes. Generally, both tissues showed a similar pattern of regulated pathways during aging. In contrast, skin showed a very heterogeneous pattern of regulated pathways across different age time points (Figure 3). It displayed no obvious pattern such as the ones seen in the brain and blood, which confirms our observations on the most significantly regulated DEGs.

Again, liver had a unique and specific pathway pattern among the four tissues. In all age group comparisons, metabolic pathways in the liver were most strongly regulated and even increased with age. A more detailed subdivision and additional details about these metabolism pathways can be found in https://osf.io/k7dba/.

However, across all regulated pathways and time comparisons, we found that the cancer-related pathways are intuitive as the interplay of cell growth promoting and tumor suppressing genes is ever-present during aging [33].

### Temporal expression profiles of tissues reveal a similar regulation of ETC in brain, blood, skin, and liver

**Figure 4:**
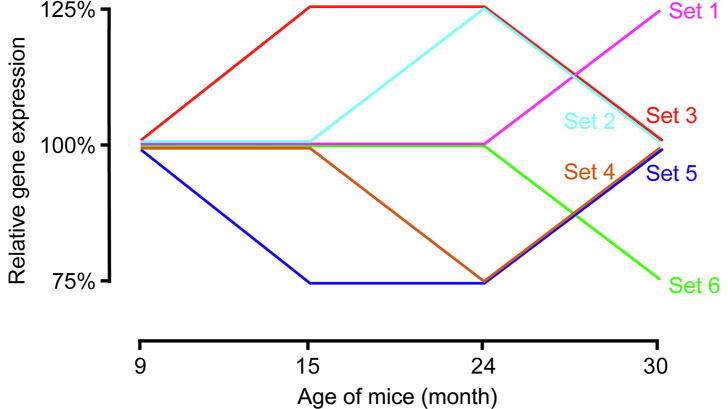
**Gene expression profiles of interest** All genes that followed the course of one of the depicted expression changes during aging were grouped into separate clusters and analyzed subsequently for an enrichment of functional commonalities. The different colored paths mark the different patterns of expression changes.

Next, we clustered all genes according to their temporal expression behaviors in the four tissues separately and analyzed specific clusters, namely, all genes that showed constant expression at earlier time points but an up- or down-regulation of at least 25% (blood, liver, and skin) or 10% (brain) at later ages. This mainly included three types of expression profiles: 1) genes that had relatively constant expression until the age of 24 months, with increased or decreased expression at 30 months, 2) genes that showed constant expression until 15 months of age, with their expression levels rising or dropping at 24 months and 30 months, and 3) genes that had increased or decreased expression at 15 months, then stayed constant until the age of 24 months, finally returned to their initial expression levels at 30 months (Figure 4). Functional annotation of these genes revealed an enrichment of genes acting in age-related KEGG pathways, such as Alzheimer’s disease, Parkinson’s disease, Huntington’s disease, non-alcoholic fatty liver disease (NAFLD), and oxidative phosphorylation in all tissues (for details visit https://osf.io/4fe3r/). A closer look yielded three interesting observations. First, all age-related pathways mentioned above shared a number of genes coding for ETC subunits in mitochondria. Second, genes related to these pathways showed higher expression at 30 months than at 24 months in every tissue, except for blood where the age of 24 months marked the highest gene expression. Third, most of these genes were related to mitochondrial dysfunction (for details visit https://osf.io/4vpzs/). These genes, including *ATP-synthetases*, *NADH Ubiquinone Oxidoreductases* (*NDUFs*), and *Cytochrome C oxidases* (*COXs*), are all part of the respiratory chain. Therefore, they are functionally related to each other, which may explain their similar expression patterns. We concluded that several complexes of the respiratory chain were similarly regulated in all four tissues during aging. A number of studies have shown that defective mitochondria, as a source of ROS, contribute to neurodegenerative disease and to aging in general, and that the suppression of complexes I, III, and IV of the ETC at a young age can increase overall life span [34], [35]. For example, Kowang and Sohal [36] examined the mitochondria isolated from brain, heart, skeletal muscle, liver, and kidney of mice and found that individual complexes of the ETC were decreased in old animals, with potentially adverse effects on oxidative phosphorylation. With increasing age, defective mitochondria in post-mitotic cells also increased in size, making it more difficult for them to undergo mitophagy. This could explain the reduced expression of the respiratory complexes in blood compared to other tissues, since it has a much higher cell turnover rate. In contrast, a previous study showed that down-regulation of ETC complexes, due to arsenic poisoning, could inhibit aerobic respiration, which can be deleterious, especially for neural tissues. Additionally, defects in succinate dehydrogenase (SDHD, complex II) can induce tumors [37]. The maintenance of optimal expression levels of the ETC genes is crucial for each tissue, in order to generate sufficient ATP from aerobic transpiration without the excessive production of ROS. Since this is a cross-sectional study, we cannot comment on the exact expression levels in the long-lived mice, leaving the question open whether mice reaching 30 months of age have a more balanced and efficient regulation of the ETC during their younger years.

### Tissue-independent markers for aging

#### Processes regulated with aging among all tissues

The striking finding of our pathway enrichment analysis showed that every tissue follows its own pattern of aging, driven by tissue-specific processes. However, we also identified processes regulated across all investigated tissues, even though regulated by different genes. In the 9 months *vs.* 30 months comparison, we found that the hematopoietic cell lineage pathway has been significantly enriched in all four tissues; however, this is due to an enrichment of inflammation-related genes, such as those encoding interleukin receptors (*e.g.*, *Il7r* and *Il4ra*) and histocompatibility complexes (*e.g.*, *H2-be1*). Additionally, several pathways related to immune response and metabolism showed enrichment in three (blood, brain, and liver) of the four tissues (Table 1). Both findings support the concept of inflammaging, as hematopoietic aging is also associated with a change in the microenvironment due to infection and inflammation [38].

**Table 1:** KEGG pathway enrichment analysis based on the genes being differentially expressed with age

*Note*: KEGG pathways that were significantly regulated in at least three of the investigated tissues were shown. *P* values were calculated based on a hypergeometrical approach used by DAVID 6.8 and adjusted using the Benjamini–Hochberg FDR approach [8]. The first two pathways are potential marker pathways for aging. No overlap was found with any enriched pathway in skin tissue.

Table 1 shows all significantly enriched pathways that were shared by the blood, brain, and liver. We determined that phagosomal pathway is one of the most affected pathways throughout aging, which again is evidence that autophagy might be one of the main driving factors of aging.

Interestingly, the cell adhesion molecule pathway seemed also to be highly affected in all three tissues during aging. Cell adhesion molecules play a crucial role in a variety of biological processes related to homeostasis, including immune response, synapse formation, and oxidative stress [39], [40]. Very likely, all other shared enriched pathways were due to their relation to inflammatory processes or immune system in general, and many of the DEGs in the investigated tissues were immune or proinflammatory genes. It is conceivable that many of these are a consequence of aging rather than a cause of it.

Nevertheless, the role of inflammation and immunity as stressors for age-related diseases and processes is actively being discussed [41], [42]. According to the suggested inflammaging phenomenon (or *inflammation hypothesis of aging*), the production of proinflammatory factors becomes less useful with age due to the accumulation of frail immune gene variants and an overall loss in specificity and efficiency of the immune system. The result is a constant inflammatory stimulus (or stress), promoting age-related processes/diseases, such as Alzheimer’s disease, osteoporosis, and diabetes. This reasoning follows the same principal ideas of the antagonistic pleiotropy hypothesis that was proposed in 1957 to explain the contrary effects of senescence and has already been the subject of aging-related discussions [43], [44]. Our data support these ideas since many of the identified alterations in gene expression are related to the immune response, infectious diseases, and/or inflammation. It remains to be elucidated what specific influence these genes might have on the aging of different tissues.

#### Commonly expressed DEGs in all tissues

**Figure 5:**
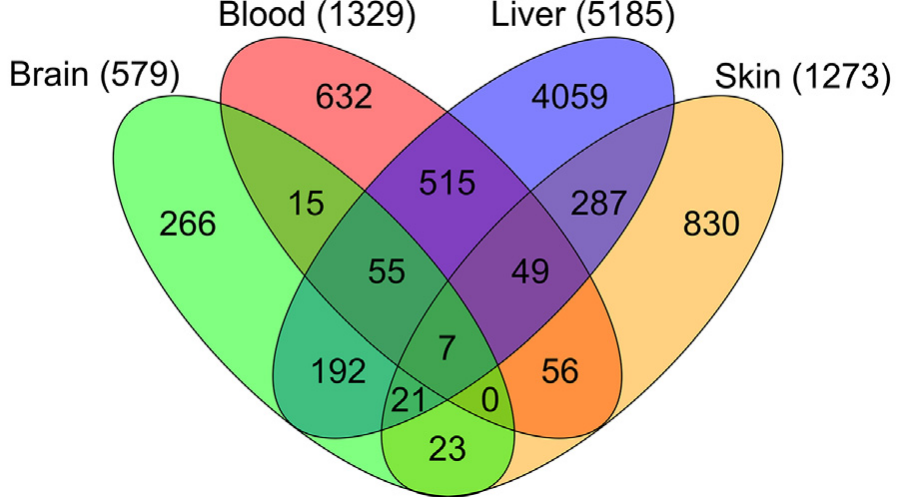
**Venn diagram of all DEGs in the four analyzed tissues** Whereas most DEGs were unique to a single tissue, a total of 125 genes were still common in at least three tissues, and seven were common in all four tissues. The total numbers of DEGs in individual tissues are written in brackets. Details and gene names can be observed in https://osf.io/uqsdc/.

Most of the DEGs were unique to a certain tissue. However, we aimed to focus on potential marker genes for aging in mouse. The overlap of all DEGs is depicted in Figure 5 (for details visit https://osf.io/uqsdc/). We found that 125 genes were common in at least three examined tissues and seven genes showed a significant change in all four tissues.

The liver showed the highest number of DEGs (5185 genes), most of which (~ 78%) were unique to the liver during aging. Analysis of the skin exposed ~ 65% unique genes, followed by the blood (~ 48%) and brain (~ 46%).

**Figure 6:**
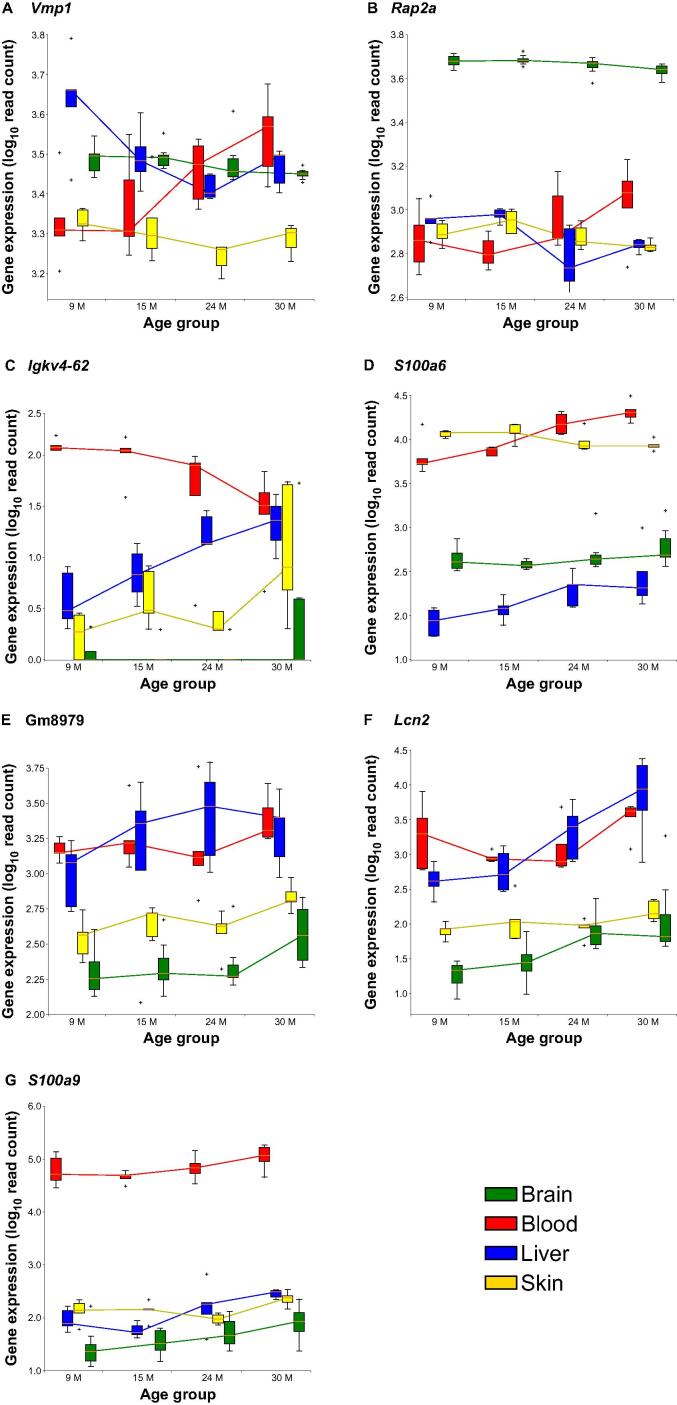
**Temporal expression profiles of the seven genes differentially expressed in all four tissues during aging** The *y*-axes provide the expression strength as log_10_ normalized counts of expressed reads of each gene. The *x*-axes provide the different investigated age groups. Small plus signs indicate outliers. With the exception of *Lcn2*, *S100a9*, and Gm8979, none of the genes showed a tendency of increased expression with age in all tissues. More details regarding the expression profiles of all seven genes can be found in https://osf.io/74ztu/ and https://osf.io/svfh7/.

The seven DEGs common to all four tissues were *Vmp1* (encoding Vacuole membrane protein 1), *Rap2a* (encoding Ras-related protein 2a), *Igkv4-62* (encoding Immunoglobulin kappa variable 4*-*62), *S100a6* (encoding S100 calcium binding protein A6), Gm8979 (encoding GTPase, very large interferon inducible, pseudogene 3), *Lcn2* (encoding Lipocalin-2), and *S100a9* (encoding S100 calcium binding protein A9), and the last three genes showed a tendency of increased expression with age in all four tissues (Figure 6; for more details visit https://osf.io/74ztu/).

LCN2 has been implicated to play a role in aging disorders in several other studies. For instance, its overexpression in the brain was linked with an increased insulin resistance in advanced age, resulting in obesity [45]. It has also been considered as a marker for multiple sclerosis [46] and was described to be associated with oxidative stress, inflammation, and demyelination of neurons, which in turn led to cognitive impairment [47]. The details about six other genes can be found in https://osf.io/u5cmv/.

### Effect of ***Lcn2*** ortholog knockdown in ***C. elegans*** using RNAi

**Figure 7:**
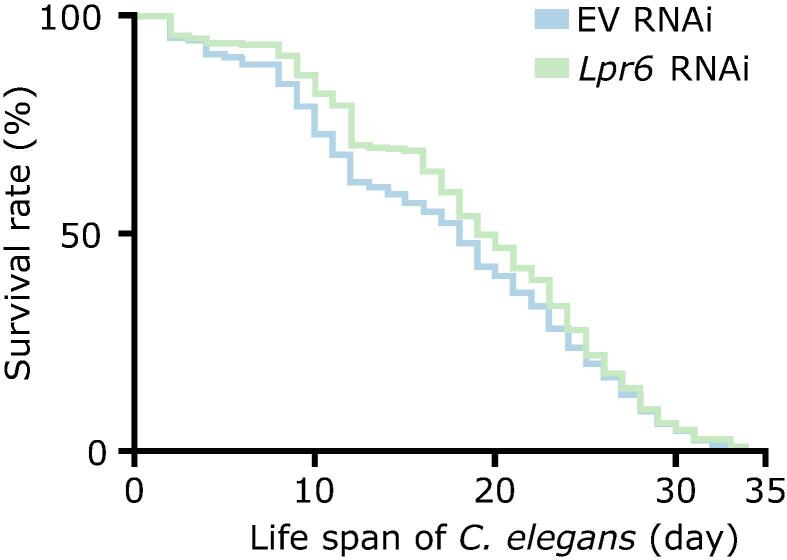
***C. elegans* survival rates with and without *Lpr6*RNAi knockdown** Survival rates of empty vector treated (EV RNAi) and *Lpr6* RNAi knockdown *C. elegans*. When compared, there seemed to be no significant increase in the total lifespan of individuals, but there was a slight protection against early lethality. The survival experiment was repeated three times (*n* = 3).

Since *Lcn2* was significantly up-regulated during aging in all samples, we further analyzed its effect on the lifespan in the invertebrate model *C. elegans* using RNAi. Lcn2 is orthologous to a family of *C. elegans* lipocalin-related proteins, which consists of seven paralogs (*Lpr1*−*7*). Only one of the paralogs, *Lpr6*, is expressed in neurons (amphid sensory neurons), suggesting that its function is probably closest to that of mammalian *Lcn2* [48]. We next decided to investigate the impact of *Lpr6* on longevity using an RNAi-mediated gene inactivation approach. As shown in Figure 7, *Lpr6* deficient animals showed a trend of extended longevity early in life, resulting in a median life span extension of one day (from 18 days to 19 days), while maximal longevity is unchanged. This suggests that down-regulation of *Lpr6* (or *Lnc2*) alone is not sufficient to have a significant influence on the overall life span, which is plausible considering the complexity of aging. However, its reduced expression still has a positive and reproducible effect on the survival rate of *C. elegans* even if it is minor.

## Conclusion

In this extensive study of 92 male murine RNA-seq libraries of four tissues at four age points, we showed that tissue-specific gene expression changes prevailed over tissue-independent aging-linked gene expression changes (Figure 1). This phenomenon indicates that different tissues age differently because of intrinsic and extrinsic factors specific to these tissues. The liver displayed a much higher number of DEGs, suggesting one of two possibilities: this might indicate better adaption to changes over time to coordinate its functions within the aging body, or on the contrary, it could also be a sign of loss of plasticity with aging. In addition, the liver has to deal with systemic toxins which become more abundant with aging among other things causing cell death and inflammation. Early on, the liver showed signs of inflammation that might later account for dysfunction of hematopoiesis in the liver [49].

On the other hand, anti-ROS-related pathways could work to sustain the normal functioning of the body. With a further increase in age, the liver showed tremendous changes in metabolic pathways, which can be considered as a two-way response to aging, *i.e.*, following and resisting certain aging processes. In addition, anti-ROS pathways are important defense mechanisms against the rise of the cancer-related pathways when comparing young and old mice [50], [51].

While investigating genes with a distinct pattern of expression during aging, *i.e.*, genes showing more drastic changes at the later time points, we found that many genes of the COX and NDUF families were regulated similarly in all four tissues. Members of the NDUF family are part of complex I of the mitochondrial ETC and have been studied prominently with respect to cancer, ROS, and lifespan [52], [53]. Being the last enzyme of the mitochondrial respiratory chain, COX plays a key role in senescence. Therefore, its respective subunit genes are eminently studied with respect to aging and cancer, and are often used to evaluate the level of oxidative stress [54], [55]. The stability of the COX assembly requires efficient ATP-synthetase activity, and we showed that the expression of ATP-synthetase genes followed the same temporal profiles as the COX genes (*e.g.*, *Cox7a2*, *Cox4i1*, and *Cox8a*), indicating that an increase in the COX expression level could be a consequence of or an effort to balance disrupted ATP-synthetase [56].

Looking at the general picture of all age affected pathways for the investigated tissues (Figure 3), we suspected chronic inflammation to be the major or at least the most prominent correlative of aging, in regard to gene expression changes. Nevertheless, the liver seems to oppose the resulting negative changes through metabolic adaptations.

The hematopoietic cell lineage pathway was the only pathway shared by all four tissues when comparing adult mice of 9 months or 15 months to a highly aged individual of 30 months, because it contains a number of inflammatory genes, which are not specific to blood. This supports the hypothesis of the so-called immunosenescence. It also underlines the fact that several inflammation-related pathways were common in at least three tissues, which further supports the theory that immunosenescence results in inflammaging [57].

We were able to identify specific DEGs that were common in all or at least three tissues. These genes could be of special interest for further aging research to determine whether they constitute a part of the aging process or result from it. The seven common DEGs in all investigated tissues are particularly important since they are involved in the immune system, cell cycle, autophagy, and calcium signaling. These are processes that are intermittently being discussed as the main cause of aging [32].

Several studies have also shown a change in the cell type population with aging, which may also affect the expression pattern of the whole tissue [58]. Therefore, it would be interesting to study the transcriptome on the single cell level to evaluate the contribution of such cell population changes with aging. Additionally, male mice were chosen in this study to avoid any interference of hormonal cycles of female mice on expression pattern of genes. However, including female mice could be helpful in understanding the process of aging in species per se and also to study sex linked signatures of aging.

In a similarly exhaustive study, based on microarray data and histological examination, Jonker et al. [1] compared five different tissues (liver, kidney, spleen, lung, and brain) from mice at five age points (13, 26, 52, 78, 104, and 130 weeks) [1]. They found several hallmark age-related pathologies across tissues and observed some tissue-specific effects, such as an accumulation of lipofuscin in brain and liver, thickening of glomerular membrane in kidney, and increased peribronchiolar lymphoid proliferation in lung. They also found *Lilrb4* (encoding Leukocyte immunoglobulin like receptor B4) to be up-regulated in all tissues. However, immune-related genes were down-regulated in spleen but up-regulated in kidney and lung which can be justified as they observed reduction in lymphocytolysis in spleen.

We conclude that aging is a very heterogeneous process, and any single gene can hardly be regarded as a marker of aging for a whole organism. Every tissue has its own specialized function. We showed and justified several tissue-specific processes to be regulated during aging. They may overlap in some cases of particular genes or common processes. However, it is difficult to identify any process or gene to be responsible for cumulative aging except for chronic inflammation and imbalances of the mitochondrial ETC. Together, the observation that most of the identified DEGs can only be found in long-term comparisons suggests that aging is a more subtle process, manifesting gradually in animals.

## Materials and methods

### Tissue and RNA preparation and RNA-seq

The male C57BL/6 mice used in the study were sacrificed at 9, 15, 24, and 30 months, respectively, followed by tissue sampling of the brain (8 replicates for each time point), blood (5 replicates for each time point), skin (5 replicates for each time point), and liver (5 replicates for each time point). Sample collection and preparation and high-throughput RNA-seq have been mentioned by Irizar et al. [59] and Frahm et al. [12] in previous publications, and detailed information can be found in https://osf.io/rhm39/. Breeding and housing conditions were as described by Mansfeld and colleagues [60].

### RNA-seq library processing and mapping

Read quality was monitored using FastQC (v0.11.3; http://www.bioinformatics.bbsrc.ac.uk/projects/fastqc/) (for details visit https://osf.io/ge8b3/). Mapping onto the current *Mus musculus* genome (GRCm38.78) was performed with segemehl (v0.2.0) [61] using the –H 1 option, allowing single reads to be mapped to multiple best fitting locations. Reads were counted using rnacounter (v1.5.2; https://github.com/delafont/rnacounter/) and normalized by transcripts per million (TPM).

$${\mathrm{TPM}}_{i}=\frac{c_{i}}{l_{i}}∙\left(\frac{1}{\sum_{j\in N}\frac{c_{j}}{l_{j}}}\right)∙10^{6}$$

where *c_i_* is the raw read count of gene *i*, *l_i_* is the cumulative exon length of gene *i*, and *N* is the number of all genes in the given annotation. Low expressed genes were defined by showing a TPM ≤ 0.5 in every sample and were discarded in subsequent expression analysis.

### **Identification of DEGs**

The Bioconductor packages DESeq2 (v1.10.0) [62] and edgeR (v3.1.2) [63] were used to identify DEGs between the analyzed time points. Half of the comparisons can be seen as a linear age-gradient analysis (9 months *vs*. 15 months, 15 months *vs*. 24 months, and 24 months *vs*. 30 months), providing insight into the changes in gene expression over time. The other three comparisons (9 months *vs*. 24 months, 9 months *vs*. 30 months, 15 months *vs*. 30 months) showed the more drastic transcriptional changes between adult and old-aged mice. The resulting *P* values were adjusted using the Benjamini–Hochberg false discovery rate (FDR) approach [64]. Genes with an adjusted *P* value < 0.05 identified by at least one of the Bioconductor packages in one of the above-mentioned comparisons were assigned as differentially expressed. Detailed information about all DEG results of both used tools and their overlap can be found in https://osf.io/k79tp/ and https://osf.io/erd85/.

### Age profiling of tissue gene expression

Each expressed gene with TPM > 0.5 was assigned an age profile with respect to its expression behavior over time for each of the four tissues individually.

The profiles were determined by analyzing the read fold change between the linear age progression comparisons (9 months *vs*. 15 months, 15 months *vs*. 24 months, and 24 months *vs*. 30 months). For each of the three comparisons, every gene was categorized either up/down-regulated (increase/decrease of at least 25% in fold change) or equally expressed. In the brain, gene expression was more consistent because the expression activity of total neurons was reduced compared to other tissues due to no further division after birth. Thus, a fold change of 10% was assumed to have significant effects in brain. Genes were clustered regarding their expression profiles, and clusters were analyzed for KEGG pathway category enrichment. For details about the age profiling of tissue gene expression visit https://osf.io/qenp2/.

### KEGG pathway category enrichment

DEGs from each age comparison (Figure 2) were associated with the KEGG [65] pathways they are involved in, using the functional annotation analyses of DAVID (version 6.8) [66] for each tissue. All pathways were grouped on the basis of their physiological roles and functions into different categories. Categories that contained at least 5% of the DEGs of the respective comparison were: metabolism, inflammation and immune response, synapse-related pathways, muscle function, ECM, adhesion and cytoskeleton, signaling pathways, and cancer. All remaining categories were grouped into ‘Others’. This analysis was done with the DEG results from DESeq2, the DEGs from the union of DESeq2 and edgeR results, and the DEGs of the intersection from the results of both tools. The enrichment was performed based on up- and down-regulated genes separately, as well as the whole set of DEGs (for details visit https://osf.io/h6dx3/ and https://osf.io/k7dba/).

Additionally, genes that followed a specific expression pattern with aging were separately used for pathway enrichment analysis (for details visit https://osf.io/4fe3r/ and https://osf.io/4bvm5/). Pathways with a FDR adjusted *P* value lower than 0.05 were considered significantly regulated and further classified into functional groups for each tissue individually.

### RNAi treatments and lifespan analysis

RNAi treatments and lifespan analysis were performed as per mentioned by Heinze et al. [67] (for details visit https://osf.io/rhm39/). The analysis of the lifespan data, including statistical analysis, was performed using GraphPad Prism software (for more details visit https://osf.io/24q79/).

## Ethical statement

All animal procedures were approved by the local government (Thueringer Landesamt, Bad Langensalza, Germany) and conformed to international guidelines on the ethical use of animals.

## Data availability

All RNA-seq data have been deposited in the Gene Expression Omnibus (GEO: GSE75192), and are publicly accessible at https://www.ncbi.nlm.nih.gov/geo/.

## Competing interests

The authors have declared no competing interests.

## CRediT author statement

**Akash Srivastava:** Conceptualization, Validation, Formal analysis, Investigation, Writing - original draft, Writing - review & editing. **Emanuel Barth:** Conceptualization, Software, Formal analysis, Investigation, Data curation, Writing - original draft, Writing - review & editing, Visualization. **Maria A. Ermolaeva:** Validation, Formal analysis, Resources, Writing - review & editing. **Madlen Guenther:** Validation, Writing - review & editing. **Christiane Frahm:** Formal analysis, Writing - review & editing. **Manja Marz:** Conceptualization, Writing - review & editing, Project administration, Funding acquisition. **Otto W. Witte:** Conceptualization, Writing - review & editing, Project administration, Funding acquisition. All authors read and approved the final manuscript.
